# Exploring Gene Expression Patterns in Alzheimer’s Disease Using a Human Microarray Data Meta-Analysis

**DOI:** 10.3390/biology15040345

**Published:** 2026-02-16

**Authors:** Eleni Dermitzaki, Vasileios L. Zogopoulos, Apostolos Malatras, Vasiliki Georgopoulou, Petrina-Marina Aslanoglou, Adamantia Teta, Maria Rea Kalligianni, Christos Karoussiotis, Vassiliki A. Iconomidou, Ioannis Sotiropoulos, Ioannis Michalopoulos

**Affiliations:** 1 Center of Systems Biology, Biomedical Research Foundation, Academy of Athens, 11527 Athens, Greece; eleniderm@tamu.edu (E.D.); vzogopoulos@bioacademy.gr (V.L.Z.); vasiliki.georgopoulou@acg.edu (V.G.); aslanoglou@fleming.gr (P.-M.A.); mandoteta@gmail.com (A.T.); reamaria.rmkalli@gmail.com (M.R.K.); 2 Section of Cell Biology and Biophysics, Department of Biology, National and Kapodistrian University of Athens, 15701 Athens, Greece; veconom@biol.uoa.gr; 3 Artie McFerrin Department of Chemical Engineering, Texas A&M University, College Station, TX 77843, USA; 4 biobank.cy Center of Excellence in Biobanking and Biomedical Research, University of Cyprus, 2109 Nicosia, Cyprus; malatras.apostolos@ucy.ac.cy; 5 Division of Animal and Human Physiology, Department of Biology, National and Kapodistrian University of Athens, 15784 Athens, Greece; 6 Department of Biochemistry and Biotechnology, University of Thessaly, 41500 Larissa, Greece; 7 Institute for Bioinnovation, Biomedical Sciences Research Center “Alexander Fleming”, 16672 Vari, Greece; 8 Department of Computer Science and Biomedical Informatics, University of Thessaly, 35131 Lamia, Greece; 9 Department of Hygiene, Epidemiology and Medical Statistics, Medical School, National and Kapodistrian University of Athens, 11527 Athens, Greece; 10 Laboratory of Brain Exosomes and Pathology, Institute of Biosciences and Applications, NCSR Demokritos, 15341 Agia Paraskevi, Greece; ckaroussiotis@bio.demokritos.gr (C.K.); ioannis@bio.demokritos.gr (I.S.)

**Keywords:** aging, differential gene expression, neurodegeneration, transcriptomics, dementia, robust multichip analysis, systems biology, brain, amyloid beta, Tau protein

## Abstract

Alzheimer’s disease (AD) is the most common neurodegenerative disorder worldwide, for which aging represents the main risk factor. As the global elderly population expands, the prevalence of Alzheimer’s disease escalates rapidly. Notably, as AD brain lesions may start 15–20 years before the appearance of the first symptoms, early diagnosis or prognosis of AD is of paramount importance for better patient treatment. Based on the absence of effective cure or early diagnosis of AD, this meta-analysis investigates the differentially expressed genes between Alzheimer’s and a healthy brain and identifies genes that can serve as risk factors for the disease or biomarkers of diagnostic, prognostic, or pharmacological value. Microarray datasets were collected from public repositories, following the Preferred Reporting Items for Systematic Reviews and Meta-Analyses (PRISMA 2020) guidelines. Quality control and data normalization were performed. Differentially expressed gene (DEG) lists were created for each study and combined through a Mosteller–Bush meta-analysis, resulting in a final list of DEGs. This list was filtered using an adjusted *p*-value cut-off of 0.001, and the included statistically significant DEGs were subjected to enrichment analyses. A total of eight microarray studies were identified, producing a combined list of 4218 DEGs, of which 1944 were up-regulated and enriched for immune response processes, and 2274 were down-regulated and enriched for synapse-related pathways. This meta-analysis reveals a distinct transcriptomic profile in Alzheimer’s disease characterized by the prevalence of immune response and inflammation alongside the collapse of essential synaptic and neuronal signaling.

## Simple Summary

Alzheimer’s disease (AD) is a neurodegenerative disorder that progressively damages the brain; however, there is currently no cure or easy way to diagnose AD early. This study aims to discover specific differences in gene activity in the brain of AD and identify genes that can serve as risk factors for disease or biomarkers of diagnostic, prognostic, or pharmacological value. Through the re-analysis and meta-analysis of data from multiple existing studies, a combined list of genes that have a statistically significant change in expression was produced. The results showed that genes responsible for communication between brain cells are less active in the AD brain. In contrast, genes involved in the body’s immune defense and inflammation are more active. These findings are valuable as the tracking of these specific gene changes could provide insights into earlier Alzheimer’s diagnosis and improved patient treatment.

## 1. Introduction

Alzheimer’s disease (AD) is a neurodegenerative disorder affecting the brain, characterized by the presence of extracellular amyloid plaques containing β-amyloid (Aβ) [1] and intracellular neurofibrillary tangles containing Tau proteins within the neuron cells [2]. Although memory loss is the main symptom, in elderly populations, AD can also show other cognitive impairments. Memory problems, particularly short-term memory loss, are the most common initial sign, but difficulties in speech, spatial awareness, and mental agility can also occur [3]. In addition, AD is one of the most expensive diseases worldwide, with a high impact on patients’ quality of life. By 2050, the prevalence is believed to double in Europe and triple worldwide [4].

Although AD is typically not passed down directly through family inheritance, genetics intricately contribute to the development of the condition in many individuals [5]. An individual is at risk for AD due to various factors, such as aging, sex, obesity, brain trauma, and hearing loss, while genetic factors and metabolic disorders also play an important role in the occurrence of AD. Dominantly inherited AD typically begins approximately 40 years earlier than sporadic late-onset AD [5]. It is believed that 70% of the risk for developing inherited AD is based on specific mutations in genes, such as *PSEN1* (Presenilin 1), *PSEN2* (Presenilin 2), and *APP* (Amyloid Beta Precursor Protein), while *APOE* (Apolipoprotein E) variants result in incomplete penetrance, with the ε4 allele being related to a higher risk [6].

In more detail, *APP* plays a crucial role in AD pathogenesis, being especially significant in individuals with Down syndrome due to the location of the gene on chromosome 21 [7]. APP undergoes proteolytic processing, primarily by ADAM10 (ADAM Metallopeptidase Domain 10) α-secretase, resulting in the formation of beneficial soluble APPα (sAPPα) peptides [8]. This peptide supports synaptic function, neuronal survival, plasticity, and stem cell proliferation, contributing to increased cognitive impairment [9]. However, APP can also be cleaved by β-secretase and γ-secretase, yielding Aβ peptides, such as Aβ40 and Aβ42, with the latter being associated with neurotoxicity. The γ-secretase complex is formed from PSEN1, PSEN2, NCSTN (Nicastrin), APH1A (Aph-1 Homolog A, Gamma-Secretase Subunit), and PSENEN (Presenilin Enhancer, Gamma-Secretase Subunit). Pathogenic Aβ accumulation disrupts various neuronal processes, leading to neurodegeneration and, ultimately, cell death. Elevated Aβ42/Aβ40 ratios are observed in AD patients [10], indicating disease progression. Aβ accumulation in the hippocampus, amygdala, and cortex triggers astrocyte and microglial activation and damages axons and dendrites, ultimately causing synapse loss and cognitive decline [11,12,13].

On the other hand, AD brain pathology also involves the formation of neurofibrillary tangles, primarily composed of hyperphosphorylated MAPT (Microtubule-Associated Protein Tau). In the AD brain, Tau protein undergoes abnormal phosphorylation at over 30 sites, facilitated by kinases, like GSK3A (Glycogen Synthase Kinase 3 Alpha), GSK3B (Glycogen Synthase Kinase 3 Beta), and CDK5 (Cyclin Dependent Kinase 5), alongside PKCs (Protein Kinase C), PKA complex, and MAPK1 (Mitogen-Activated Protein Kinase 1). Tau hyperphosphorylation leads to its malfunction, leading to Tau oligomerization and aggregation into helical fibrils, destabilizing microtubules and causing deficits of different neuronal functions, including synaptic function and axonal transport that affects intraneuronal localization of different molecules and/or organelles, such as mitochondria [14,15]. Notably, under normal conditions, Tau protein is involved in mitochondrial function, with a specific localization in mitochondrial membranes, suggesting intracellular movement to regions with high calcium concentration, such as axons and dendrites. However, in the AD brain, mitochondria are found in neuronal bodies instead, while mitochondrial function is dysregulated [16].

*APOE*, crucial for cholesterol transport, significantly influences AD precipitation. Its three alleles exhibit varying effects, with the APOE-ε4 allele, especially in homozygosity, significantly increasing AD risk, potentially due to elevated cholesterol levels [17]. Also, *APOE* ε4 carriers present higher scores in verbal fluency compared with ε3/ε3 patients, possibly due to an antagonistic pleiotropic behavior [18]. High cholesterol correlates with increased Aβ production, as cholesterol-rich environments inhibit the function of ADAM10, affecting sAPPα peptide production, which has a protective role against AD [19]. In contrast, the APOE-ε2 allele exhibits a protective effect against AD [4,20,21].

Until now, there has been no sufficient treatment to block the progression of the disease or track its progression stages. Despite recent advancements in the treatment of early AD based on monoclonal antibodies against Aβ, longer and more comprehensive clinical trials are necessary to fully establish the long-term efficacy and safety profile of lecanemab in this patient population [22], while this treatment option is limited to a specific subgroup of AD patients. Furthermore, there is still a lack of highly specific biomarkers to accurately reveal the stages of the disease and enable early AD diagnosis. Thus, the main purpose of this meta-analysis is to reveal a list of genes whose expression is altered in the AD brain and may serve as potential biomarkers for diagnostic or prognostic purposes.

## 2. Materials and Methods

### 2.1. Pipeline Summary

**Figure 1:**
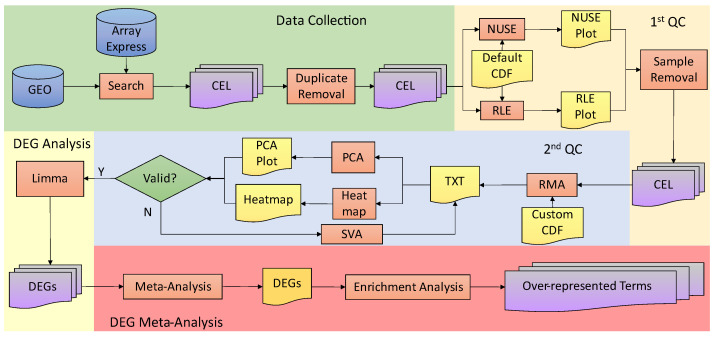
Differential gene expression analysis workflow. Data collection (from GEO and ArrayExpress), quality control of each sample in each study (NUSE and RLE), pre-processing, which includes normalization with the Robust Multi-array Average (RMA) algorithm and batch effect correction (SVA), differential gene expression analysis using the limma package, and implementation of meta-analysis by the generation of the final list. This list is used for biological term enrichment analysis.

The microarray analysis used in this study is summarized in the following flowchart representing the search process and bioinformatics pipeline (Figure 1).

### 2.2. Specification of Inclusion and Exclusion Criteria

The search for microarray datasets was performed up to 20 January 2026. The included studies were manually and individually examined to check if they met the following criteria: (1) the tissue type corresponds to brain sections and not cell lines or blood tissue; (2) they only include experiments performed with Affymetrix platform chips; (3) they contain both pathological and normal samples; (4) their condition samples are only AD; and (5) no drug assessments are tested.

### 2.3. Data Extraction

The search for publicly available microarray datasets for human AD and healthy samples was conducted using Gene Expression Omnibus (GEO) of the National Center for Biotechnology Information (NCBI) [23] and ArrayExpress of the European Molecular Biology Laboratory–European Bioinformatics Institute (EMBL-EBI) [24] public repositories, which comply with Minimal Information About Microarray Experiments (MIAME) principles [25]. To ensure reliability and transparency, we employed the Preferred Reporting Items for Systematic Reviews and Meta-Analyses (PRISMA 2020 flow diagram for new systematic reviews which included searches of databases and registers only) guidelines [26].

### 2.4. Search Strategy

The advanced search query in GEO was: ((alzheimer*[Title] OR alzheimer*[Description]) AND “expression profiling by array”[DataSet Type]) AND “Homo sapiens”[porgn] AND “gse”[Filter] AND CEL. Similarly, in the search query in ArrayExpress, the search term was “alzheimer disease”, the Study Type was set to “transcription profiling by array”, the Organisms to “homo sapiens”, and the File Type to “cel” (Table S1).

### 2.5. Selection Process

Resulting studies were extracted and classified in Microsoft Office Excel and manually checked to ascertain their compliance with the selection criteria. A Venn diagram was drawn using https://bioinformatics.psb.ugent.be/webtools/Venn/ (accessed on 20 January 2026) for the comparison between the studies resulting from each database (GEO and ArrayExpress) query in order to avoid duplicate studies with identical accession numbers.

### 2.6. Data Collection

Raw data .CEL files, which contain the intensities per probe, were downloaded from each repository. These files were converted from Binary to ASCII format (txt format), using Array Power Tools [27], and the discovery of duplicate studies/samples and their subsequent removal was performed programmatically, using custom PHP scripts. The meta-data for each study’s samples, containing patient details such as age, sex, diagnosis, tissue, and severity of the disease measured using Braak staging [28] and/or cognitive scores, were also collected when available.

### 2.7. Quality Control of Samples of Each Study

Quality control was performed for the samples of each study, with the already described criteria [29,30]. Briefly, since the included studies contained chips from different Affymetrix platforms and versions, two plots were employed to perform quality control assessments, Normalized Unscaled Standard Error (NUSE) and Relative Log Expression (RLE) [31] plots, both of which are based on the intensities per probe set after MAS5 algorithm [32] normalization per study. Samples per study that exceeded the established thresholds for these boxplots (NUSE = 1.05 ± 0.10 and RLE = 0.0 ± 0.2) were removed. NUSE and RLE plot generation was performed in R (version 4.30) using oligo [33], which belongs to the Bioconductor suite [34], and the exclusion was performed automatically using custom PHP CLI (version 7.4) scripts.

### 2.8. Normalization—Batch Correction

Studies that contained samples from more than one tissue were split into multiple sub-studies that only contained samples from the same tissue. Pre-processing was performed using the RMA algorithm [35], which is based on quantile normalization [36], coupled with the latest version of each CDF (Chip Description File) procured from BrainArray [37]. This ensured that there was a 1-to-1 correspondence between probe sets and genes. For every study, a gene expression matrix of all samples was produced.

To visually check for possible batch effects and/or low-quality samples that passed the automatic quality control, heatmaps were generated for each study to investigate gene expression motifs [38] between the two conditions (AD and healthy). Also, Principal Component Analysis (PCA) plots were generated in order to cluster the samples into two distinct categories (AD and healthy). The creation of the categories is based on the expression motifs for each sample in a study, reflecting the similarities between the two conditions [39].

Additionally, to minimize the variability among the studies, batch effect correction was performed in each study if it was deemed necessary. This variability can derive from the different regions of brain tissue, different laboratory protocols across labs, scanners, etc. [40]. The SVA algorithm [41] was applied for the minimization of these technical biases that resulted in batch effect, and the batch-corrected gene expression matrices for each study were once again visually checked using PCA plots.

### 2.9. Differential Gene Expression Analysis

Differential expression analysis was performed using limma [42] to produce lists of differentially expressed genes (DEGs) for each study in the analysis. Using the org.Hs.eg.db package [43], genes were further annotated with their corresponding HGNC [44] gene symbol and name. The exported gene lists in each study contain the log_2_ fold change, *p*-value, and Benjamini–Hochberg (BH) [45] false discovery rate (FDR)-adjusted *p*-value (adjP) for each gene.

### 2.10. Statistical Meta-Analysis

The DEG lists were further combined in a meta-analysis to reveal genes that are differentially expressed across all studies, as previously performed [46,47]. Briefly, our meta-analysis combined the *p*-values of every study (or every sub-study, if a study contains samples from multiple tissues) for each gene, using the Mosteller–Bush approach [48], which is a weighted variant of classic Stouffer [49], as it takes into consideration the number of samples included in a study. For each gene in every study, the two-tailed *p*-value was transformed into a one-tailed *p*-value, according to the sign of the log_2_ fold change. For each one-tailed *p*-value, a *z*-score was calculated, using the inverse normal distribution $\Phi^{-1}$, in Equation (1):

(1) $$z_{i}=\Phi^{-1}\left(p_{i}\right)$$

where $p_{i}$ is a gene’s one-tailed *p*-value in study *i* and $z_{i}$ is its z-score. A Mosteller–Bush-based z-score was calculated by Equation (2):

(2) $${\;z}_{mb}=\frac{\sum_{i=1}^{k}\left(n_{i}-2\right)z_{i}}{\sqrt{\sum_{i=1}^{k}{\left(n_{i}-2\right)}^{2}}}$$

where $z_{mb}$ is the meta-analysis z-score for each gene, $n_{i}$ is the samples of the study *i*, and *k* is the number of all studies. For each meta-analysis *z*-score, a one-tailed *p*-value was calculated, using the normal distribution Φ, in Equation (3):

(3) $$p_{mb}=\Phi (z_{mb})$$

where $p_{mb}$ is the meta-analysis of the one-tailed *p*-value. Subsequently, the one-tailed *p*-values were converted to two-tailed *p*-values. Finally, *p*-values underwent FDR adjustment to produce an adjusted *p*-value, with 0.001 being selected as the significance cut-off. The sign of the z-score indicates whether a DEG is over- or under-expressed.

### 2.11. Enrichment Analysis

WebGestalt 2024 [50] was used to perform enrichment analysis on the up- and down-regulated DEGs. Over-representation analysis (ORA) was selected to find which biological terms are prevalent in those two gene sets. These terms include all aspects of Gene Ontology (Biological Process, Cellular Component, and Molecular Function) [51], biological pathways (KEGG and Reactome) [52,53], network (transcript Factor target and microRNA target) [54,55], disease (DisGeNET) [56], and chromosomal location (CytogeneticBand). For the WebGestalt analysis, the union of the genes of the different Affymetrix platforms of the studies included in the meta-analysis, based on the custom BrainArray version 25 CDFs, was used as the background gene list (Data S1). In addition, protein–protein interaction (PPI) network construction, for the up- and down-regulated genes, respectively, was performed using STRING v12 [57], with default settings. Hub genes for the PPI networks were identified as the genes with the most interactions. For large PPI networks (>2000 input genes), the Cytoscape stringApp v2.2.0 [58] was used, and hub genes were calculated using the internal Cytoscape 3.10.4 analysis tools [59].

## 3. Results

### 3.1. Database Search

**Figure 2:**
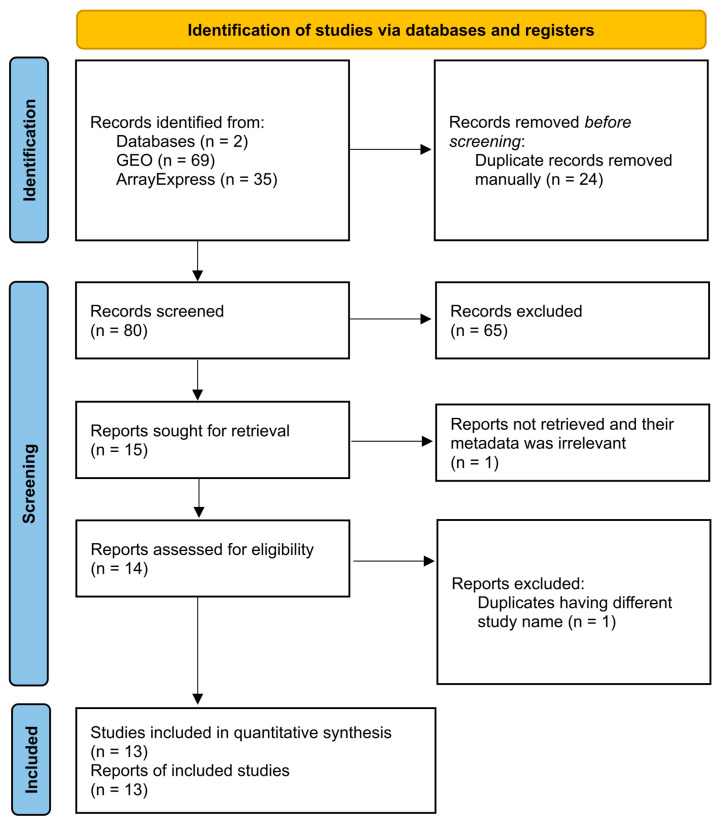
PRISMA 2020 flowchart depicting the strategy of the initial online repository search.

Data selection was performed following the PRISMA 2020 guidelines (Figure 2). The ArrayExpress search resulted in 35 studies, and the GEO search resulted in 69 studies. Out of those 104 studies, 24 ArrayExpress studies were excluded, as they were found to be duplicates of GEO studies. Out of the 80 remaining studies, 65 were removed by reading the title and abstract. Out of the 15 remaining studies, GSE28146 [60] was excluded after screening the meta-data, as the samples were stored in paraffin blocks, and GSE37264 [61] was removed as it was a duplicate of GSE37263 [62]. Finally, the following 13 studies were included: GSE39420 [63], GSE48350 [64], GSE36980 [65], GSE26972 [66], GSE37263 [62], GSE16759 [67], E-MEXP-2280 [68], GSE12685 [69], GSE1297 [70], GSE93885 [71], GSE195872 [72], GSE150696 [73], and GSE5281 [74,75] (Table S2).

Samples GSM1176197, GSM1176215, GSM1176233, and GSM117625 were removed from GSE48350, as they were identical to GSM300181, GSM300182, GSM300183, and GSM350078, respectively, with the latter ones being kept.

### 3.2. Quality Control

Quality control was performed, and samples not complying with the NUSE and RLE plot thresholds were automatically removed. In GSE93885, all control samples were removed, as they did not surpass the quality thresholds, leading to the study’s exclusion. Studies GSE150696 and GSE195872 were also removed, as not all brain samples passed quality control (Table S2). GSE26972 was excluded, as quality control failed to run. GSE37263 was also rejected, as no BrainArray custom CDF was available for its exon microarray platform (huex10st.v2). As a result, eight studies remained for the quantitative analysis (meta-analysis), and each sample’s meta-data were also collected (Data S2).

### 3.3. Differential Expression Meta-Analysis

First, the RMA algorithm was applied separately for each tissue sub-study. PCA 3D plots were created for all studies/sub-studies, with samples being removed from some sub-studies by manual inspection (Table S3). SVA batch correction was then run in all cases, and PCA plots were created again where SVA was executed successfully (Figure S1, Table S4). In GSE36980, only samples that were documented as “AD”, “AD-like change”, or “No Dementia” were used. From GSE5281, only samples from the Primary Visual Cortex and Posterior Cingulate Cortex tissues were retained, as those tissues had at least three control samples, while the Primary Visual Cortex samples were eventually deleted, as primary sensory areas do not suffer the same transcriptional disruption compared to the temporal and frontal lobes [76]. Finally, to reduce the weight of GSE48350 that contained over 200 samples, only one out of the four sub-studies was retained containing hippocampus samples, as among the two regions initially affected by AD (entorhinal cortex and hippocampus) [77,78], the hippocampus samples were less than those of the entorhinal cortex (29 vs. 36). Finally, 10 sub-studies were included, originating from eight studies (Table 1).

DEG analysis was performed for the samples of each sub-study through limma. Regarding the meta-analysis, the DEG lists from these 10 studies/sub-studies were combined to produce a final list of statistically significant DEGs for AD using a 0.001 adjP cut-off containing 4218 genes. Of them, 1944 were up-regulated (Data S3) while 2274 were down-regulated (Data S3).

The biological term over-representation analysis of the up-regulated DEGs (Table 2) identified Gene Ontology Biological Process (GO:BP) terms, all having <10^−10^ adjP, which are mainly related to immune response. Those terms include the main term of “immune response” as well as its regulation, cytokine production, cell population proliferation, etc. Gene Ontology Molecular Function highlighted the “protein-containing complex binding” term as enriched. Reactome also showcased “innate system process” as an affected pathway, supporting the enriched GO:BP terms. In addition, transcription factors belonging to the families of IRF, ETS, NF-κB, etc., were found to regulate the expression of the up-regulated genes. Finally, DisGeNET showed the “IgA glomerulonephritis” disease as enriched.

**Figure 3:**
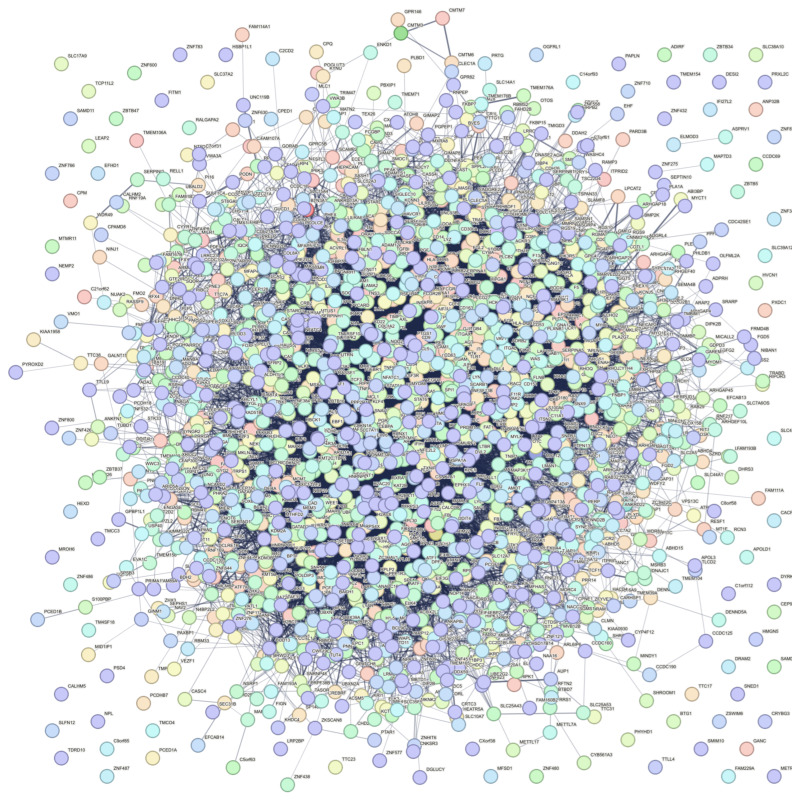
STRING-based PPI network using the up-regulated DEGs as inputs. Of the 1944 genes, 1824 were mapped as protein nodes.

In addition, the resulting STRING-based PPI network (Figure 3) had 1824 protein nodes with 21,552 edges between them, while the expected number of edges was 13913, meaning that the PPI network is significantly enriched (PPI enrichment *p*-value < 1.0 × 10^−16^) compared to a random PPI of the same size, and, thus surmising a biological connection among the nodes. The hub protein of this network was MYC (MYC Proto-Oncogene, BHLH Transcription Factor) (Data S4). The STRING enriched terms of the PPI network, through its built-in enrichment analysis, also showcased similar enriched terms to those of WebGestalt.

The respective biological term enrichment analysis for the down-regulated genes (Table 3) showcased terms that were mainly related to synapses. GO:BP highlighted terms related to cellular respiration and synaptic transmission. The Gene Ontology Cellular Component enrichment results showed that the cellular components mostly affected were related to different parts of the nerve cells, such as synapses and dendrites, as well as mitochondria and vesicle membranes. Reactome, apart from the neuronal system, also revealed the citric acid cycle and respiratory electron transport pathway as enriched. In addition, transcription factors REST, SF1, ESRRA, and RFX1, among others, were found to regulate the expression of the down-regulated genes.

**Figure 4:**
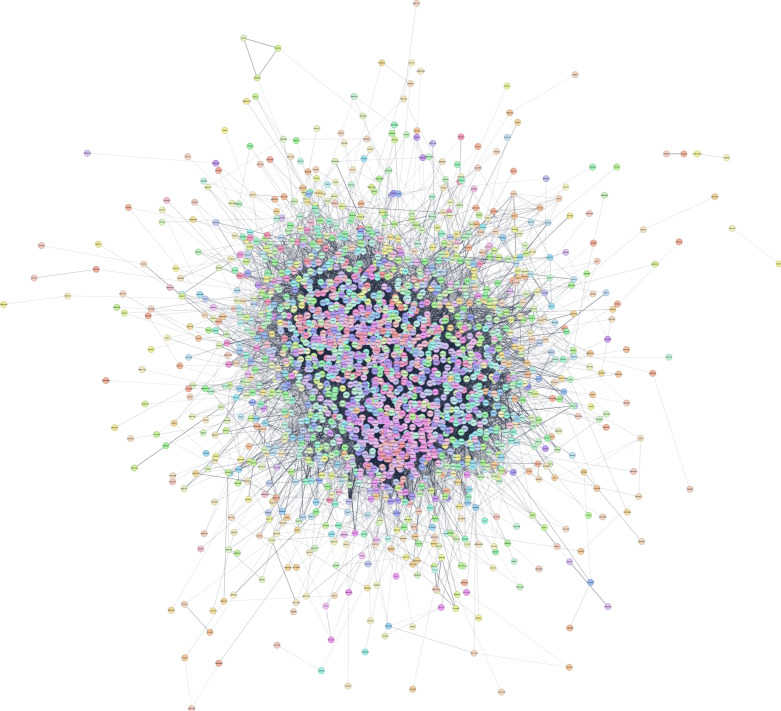
STRING-based PPI network using the down-regulated DEGs as inputs, visualized through the STRING app in Cytoscape. Of the 2274 genes, 2217 were mapped as protein nodes.

Furthermore, the resulting STRING-based PPI network in Cytoscape (Figure 4) had 2217 protein nodes with 24,958 edges between them. The hub protein of this network was GAPDH (Glyceraldehyde-3-Phosphate Dehydrogenase) (Data S4), which encodes for an enzyme essential for glycolysis. Built-in STRING enrichment analysis similarly showcased identical biological terms to those shown as enriched by WebGestalt.

## 4. Discussion

AD brain is characterized by the overproduction of Aβ and the accumulation of hyperphosphorylated Tau; both are shown to trigger synaptic malfunction and loss, as well as dendritic atrophy in neurons of different brain areas, such as the hippocampus and prefrontal cortex. Interestingly, in relation to the main AD pathomechanisms, our findings show that APP, the precursor protein of Aβ, was found not to be differentially expressed in the AD brain. Additionally, the gene of Tau, *MAPT,* is also not differentially expressed; however, GSKIP (GSK3B Interacting Protein), an inhibitor of GSK3B (Glycogen Synthase Kinase 3 Beta), is down-regulated, which may be related to increased GSK3B activity and downstream Tau hyperphosphorylation that are found in the AD brain. The top over-expressed gene of our meta-analysis, CRTAP (Cartilage Associated Protein), was shown to be associated with AD-specific Aβ pathologies [79].

Furthermore, a variety of genes are found to be differentially expressed in the AD brain in our study, with the up-regulated ones mostly related to immune response and inflammation. In addition, most of the enriched transcription factors targeting the up-regulated genes, such as IRF1 and NF-κB, control immune response. The enrichment of “IgA glomerulonephritis disease” reflects the strong representation of immune activation, cytokine signaling, and inflammatory pathways in the up-regulated genes, indicating an immune complex-mediated rather than a kidney-specific cause. In AD, B cells have a dual role, as they may worsen pathology by producing proinflammatory autoantibodies against Aβ and Tau, or they may also be protective by facilitating antibody-mediated clearance of these aggregates [80], thus explaining the prevalence of immune response terms characterizing the up-regulated genes. Indeed, neuroinflammation is increasingly recognized as a key factor in the progression of AD. Since the 1980s, studies have identified microglia and immune-related proteins in proximity to Aβ plaques [81,82]. More recent genome-wide association studies (GWASs) and PET imaging studies confirmed the involvement of microglial activation and inflammatory signaling in early AD pathology [83,84]. Inflammation is strongly associated with the risk and progression of dementia, as individuals with elevated inflammatory markers are more likely to develop dementia, and those already affected tend to decline more rapidly [78,85]. In particular, some studies suggest that anti-inflammatory treatments may reduce the risk of developing AD by up to 50% [81]. Chronic neuroinflammation in the AD brain is thought to be a reactive process due to the neuronal loss, and it is known to facilitate and exacerbate both Aβ and Tau pathologies, potentially linking early Aβ accumulation to subsequent Tau pathology [81]. Preclinical studies have shown that inflammation can lead to cognitive impairment, neuronal damage, and synaptic loss [78,86,87].

More specifically, TREM2 (Triggering Receptor Expressed on Myeloid Cells 2), a gene crucial for the brain’s immune response through DAP12 signaling, was found to be up-regulated in the AD brain. TREM2 is expressed in myeloid cells that regulate microglial functions, brain homeostasis, and affect Aβ and Tau pathologies, as well as inflammatory responses and metabolism. TREM2 can function independently or together with molecules such as APOE. Consequently, microglia enhance Aβ production in response to inflammation by releasing proinflammatory agents and up-regulating β-secretase and γ-secretase enzymes, leading to increased Aβ accumulation. In vivo data indicate that TREM2 affects Aβ deposition in a disease stage-dependent manner [88]. Recent insights into microglial functions provide a new explanation: TREM2 deficiency reduces microglial interaction with initial amyloid-β seeds, thereby decreasing seeding efficiency and delaying early-stage amyloid-β pathology. However, in the advanced AD stages where plaques are established, TREM2 is essential for plaque compaction. TREM2 deficiency impairs this function, resulting in diffuse, non-compacted plaques that exacerbate the pathology of Aβ [89]. TREM2 can be cleaved by ADAM10 (which is one of the main α-secretases), generating soluble TREM2 (sTREM2) [90]. Through a meta-analysis, it was discovered that sTREM2 levels are higher in the early stages of AD but decrease as the disease progresses to the dementia/symptomatic stage [91]. Thus, it could be used as a potential biomarker as it can be detected in CSF, determining the disease or AD stage [88].

Moreover, CLU (Clusterin), which is up-regulated in the DEG list of the current meta-analysis, is primarily produced by astrocytes and involved in lipid transport and Aβ clearance. Its expression increases with aging, stroke, and type 2 diabetes, all potential risk factors for AD. Different preclinical studies in mice indicate that major AD risk factors converge at CLU up-regulation, with CLU localizing to all neuritic plaques and remaining undetectable in glia. Consistent with in vivo findings, CLU was endogenously expressed in primary neurons. While multiple stressors increased intracellular CLU, only replicative senescence induced by prolonged culturing up-regulated both intracellular and extracellular CLU. The extracellular increase possibly reflected active secretion rather than passive accumulation, as CLU levels rapidly recovered after complete medium replacement [92]. CLU was also discovered in a GWAS as a candidate gene for LOAD (late-onset AD) [93]. Additionally, another GWAS proposed CD2AP and MS4A4A, both up-regulated in our study, as risk factors for LOAD [94], while a different GWAS proposed them as risk factors for AD [95].

On the other hand, our meta-analysis showed a set of down-regulated genes that are strongly related to synapses, with enriched terms containing synaptic structure, vesicle cycling, neurotransmission, and mitochondrial energy metabolism, indicating a coordinated suppression of neuronal communication and bioenergetic support, which is indicative of AD pathogenesis. This is also supported by the enriched neuronal-specific components, where the down-regulated proteins were localized. Finally, enriched transcription factors targeting down-regulated genes include REST, ESRRA, and RFX1. REST (RE1 Silencing Transcription Factor) encodes a transcription factor associated with repression of neurogenesis but is increasingly recognized as a critical regulator of neuronal resilience during aging [96,97]. In healthy aging, REST undergoes increased nuclear translocation in hippocampal and cortical neurons, where it suppresses pro-apoptotic and AD–associated genes [97]. Consistently, genetic deletion or down-regulation of REST in mice results in age-related neurodegeneration, impaired synaptic plasticity, cognitive decline, and activation of glial and inflammatory pathways, underscoring its essential role in maintaining neuronal homeostasis. Mechanistically, REST deficiency disrupts autophagic flux, induces mitochondrial dysfunction, and promotes a neuronal senescence phenotype, characterized by cell cycle exit, reduced viability, and SA-β-gal positivity [98,99]. In AD, the REST function is compromised through multiple mechanisms, including sequestration within autophagosomes together with misfolded Aβ and Tau, leading to reduced nuclear REST levels and widespread dysregulation of neurodegeneration-associated gene networks. Correspondingly, loss of REST accelerates Aβ and Tau pathology as well as cognitive impairment in AD mouse models, whereas hippocampal overexpression of REST suppresses these pathological features, supporting its role as a neurodegeneration checkpoint [100]. Notably, REST regulation is highly context-dependent; while elevated nuclear REST in healthy aging brains correlates with synaptic preservation, this protective response is absent in AD, and human post-mortem studies suggest complex interactions between REST expression, microRNA regulation, and disease risk [101]. Collectively, these findings position REST as an important molecular link between aging, neuronal stress resistance, and vulnerability to AD.

Moreover, we found that other genes related to synaptic homeostasis and function, such as NPTXR (Neuronal Pentraxin Receptor), were down-regulated. Indeed, NPTXR levels are found to be reduced in cerebrospinal fluid (CSF) of AD patients and are suggested as an AD biomarker [102]. The above findings are related to the characteristic neuronal atrophy (e.g., synapse loss and dendritic atrophy) of the AD brain, which is causally related to increased levels of Aβ and hyperphosphorylated Tau. Note that brain atrophy begins in the entorhinal cortex (EC) and hippocampus and spreads through the frontotemporal cortex and subsequently affects the striatum and thalamus [78].

Apart from synapses, mitochondria were also identified as an enriched cellular component characterizing the down-regulated genes. Mitochondrial dynamics are markedly perturbed by down-regulation of key fusion/fission genes in AD. For example, the fusion GTPase OPA1 and DNM1L (encoding the fission factor DRP1), discovered both as under-expressed in our study, are down-regulated in AD hippocampus, reducing mitochondrial fusion [103,104] and leading to excessive fission and fragmentation. Also, loss of OPA1 leads to smaller, fragmented mitochondria with disorganized internal folds, compromising respiratory efficiency and calcium handling in AD neurons.

Down-regulation of mitochondrial metabolic genes also impairs bioenergetics in AD. Transcriptomic analyses reveal that multiple nuclear-encoded oxidative phosphorylation (OXPHOS) subunits, such as NDUFA6 and NDUFB5, are reduced in the AD brain [105,106]. In addition, the neuron-specific carnitine palmitoyltransferase CPT1C is diminished under Aβ exposure, and its overexpression can rescue neurons from Aβ-induced reactive oxygen species (ROS) and apoptosis [107]. Thus, CPT1C down-regulation in AD likely diminishes fatty acid fuel utilization and heightens oxidative stress. Collectively, reduced expression of these metabolic genes deprives AD neurons of ATP and promotes ROS accumulation.

Mitochondrial damage and impaired mitophagy are key hallmarks of aging and neurodegeneration [108,109]. The mitophagy receptor BNIP3 is significantly repressed in the AD hippocampus [110]. Loss of BNIP3 impairs recognition of damaged mitochondria by the autophagy machinery, reducing their clearance and leading to excessive ROS and neuronal injury. Together, these findings illustrate how the down-regulation of specific mitochondrial genes disrupts energy metabolism, morphology, and mitophagy, dysregulating mitochondrial homeostasis in AD. The appearance of MRPL15 (mitochondrial ribosomal protein L15) as the most strongly down-regulated gene also points to disrupted mitochondrial translation as a contributor to mitochondrial dysfunction in AD [111]. Ultimately, disruption of mitochondrial homeostasis in AD converges on the activation of neuronal cell death pathways [112]. Down-regulation of genes controlling mitochondrial dynamics, bioenergetics, and quality control promotes accumulation of fragmented, energetically compromised mitochondria. These dysfunctional organelles exhibit reduced ATP production, impaired calcium buffering, and excessive ROS generation, all of which sensitize neurons to apoptosis. The release of mitochondrial pro-death factors, such as CYCS (Cytochrome C, Somatic), fails to eliminate damaged mitochondria through mitophagy and further exacerbates caspase activation and synaptic degeneration, ultimately driving progressive neuronal loss, which is characteristic of AD [113,114].

There are two main apoptotic mechanisms that lead to the degeneration of neurons. The first one is based on increased concentration of intracellular calcium, which relies on calpains. CAPN2 (Calpain 2) is overexpressed and is associated with AD [115]. Similarly, an activating caspase, CASP8 (Caspase 8), is up-regulated and activates CASP7 (Caspase 7), which is responsible for processes such as apoptosis. Apart from that, it also participates in the cleavage of other molecules such as APP, ROCK1 (Rho Associated Coiled-Coil Containing Protein Kinase 1), LMNA1 (Lamin A/C), and others [116,117]. Furthermore, many genes functionally related to TNF are implicated in cell death as they participate in FAS (Fas Cell Surface Death Receptor)/TNFR (TNF Receptor) pathways, activating CASP8 [118], and they were up-regulated. The second main apoptotic mechanism is linked to endoplasmic reticulum (ER) stress, a process involving the release of luminal calcium into the cytosol. This calcium efflux activates calpains, which in turn activate caspases, the primary executors of apoptosis.

**Figure 5:**
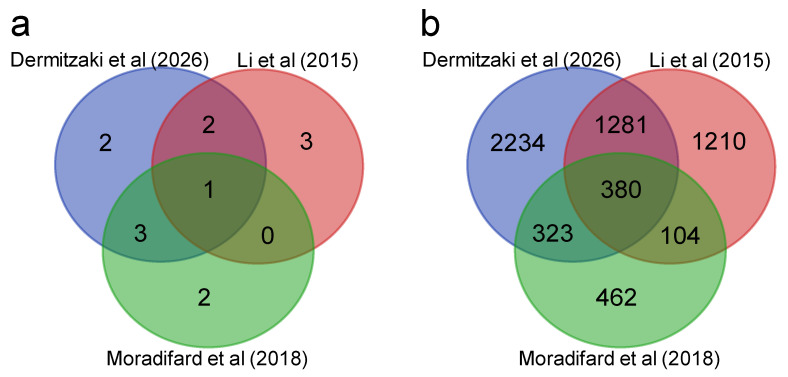
Comparison between this study and two previous microarray-based meta-analyses of AD patient brain samples [119,120]. (**a**) Common studies between the three meta-analyses; (**b**) common DEGs of the three meta-analyses.

Two previous microarray-based meta-analyses on brain samples of AD patients, each using six different studies in 2015 [119] and 2018 [120], discovered 3124 DEGs (1358 up-regulated and 1766 down-regulated) and 1414 DEGs (672 up-regulated and 742 down-regulated), respectively. To compare the common DEGs between the two previous meta-analyses and our study, g:Profiler [121] was used to map the Entrez gene IDs into ENSG IDs, removing genes that did not map into any ENSG ID, as well as duplicate ones, resulting in 2975 and 1269 unique ENSG IDs in each study, respectively. Those two lists were compared with our study’s 4218 DEG ENSG IDs, with 380 genes being common between all three meta-analyses. In addition, 1661 (~56% of the 2975) genes were common with the 2015 meta-analysis, and 703 (~50% of the 1414) were common with the 2018 one, while the two other meta-analyses had 484 (~16.2% of the 2975 and ~34.2% of the 1414) common DEGs between them (Figure 5).

Nevertheless, there are several differences between those two previous meta-analyses and ours, although in all cases, a re-analysis of the primary data was performed. While the two previous meta-analyses were both based on six studies, our own was based on eight meticulously selected studies, further divided into 10 sub-studies, increasing the statistical strength of the meta-analysis. In addition, in the two previous studies, a meta-analysis was performed using an inverse variance-weighted effect size method [122] for the 2015 study or using robust rank aggregation [123] for the 2018 one. In the 2018 study, normalization was performed using PLIER [124]. In the 2015 study, normalization was performed using RMA, coupled with the default Affymetrix CDF. In the case of a probe set corresponding to multiple genes, the probe set was removed. If multiple probe sets corresponded to one gene, the authors retained only the probe set with the largest absolute estimated effect size. On the contrary, normalization in our meta-analysis was performed using RMA coupled with the latest BrainArray CDF for each platform, not only encompassing the most recent genomic knowledge but also guaranteeing a one-to-one correspondence between probe sets and genes. This removed the need for error-prone probe set and gene eliminations. It should be emphasized that the use of default CDFs in Affymetrix studies may render up to half of identified DEGs as artifacts [37]. Moreover, in the 2015 meta-analysis, pre-filtering was based on probe set absent calls and/or low average expression in each study. On the contrary, pre-filtering in 2018 and our meta-analysis was performed based on the recommended cut-offs by Affymetrix for the NUSE and RLE plots, resulting in the selection of high-quality samples from each microarray study.

## 5. Conclusions

Our meta-analysis suggests that the AD brain suffers from a transcriptomic imbalance that includes up-regulated genes related to immune response, inflammation, and apoptotic pathways in parallel to down-regulated genes, which are essential for synaptic integrity, vesicle cycling, and mitochondrial function. This suggests that AD brain pathology is characterized by the simultaneous collapse of neuronal bioenergetics and communication, as well as the activation of calcium-mediated and endoplasmic reticulum (ER) cellular stress. Ultimately, the findings portray the brain state of AD as a condition in which aggressive neuroinflammation overwhelms the brain’s diminishing capacity for synaptic maintenance and mitochondrial energy support.

## Figures and Tables

**Table 1:** Study characteristics included in the meta-analysis.

Study	Platform	Tissue	Control Samples	AD Samples	Number of Genes	Reference
GSE48350	Affymetrix Human Genome U133 Plus 2.0 Array	Hippocampus	22	7	19,914	[64]
GSE39420	Affymetrix Human Gene 1.1 ST Array	Posterior Cingulate Cortex	4	10	21,344	[63]
GSE36980	Affymetrix Human Gene 1.0 ST Array	Hippocampus	5	4	21,369	[65]
		Frontal Cortex	6	7		
		Temporal Cortex	12	4		
GSE16759	Affymetrix Human Genome U133 Plus 2.0 Array	Parietal Lobe	3	4	19,914	[67]
GSE1297	Affymetrix Human Genome U133A Array	Hippocampus	3	7	11,733	[70]
GSE5281	Affymetrix Human Genome U133 Plus 2.0 Array	Posterior Cingulate Cortex	5	3	19,914	[74,75]
GSE12685	Affymetrix Human Genome U133A Array	Frontal Cortex	6	5	11,733	[69]
E-MEXP-2280	Affymetrix Human Genome U133 Plus 2.0 Array	Medial Temporal Lobe	3	6	19,914	[68]

**Table 2:** Representative biological terms enriched in the up-regulated DEGs, as highlighted by WebGestalt ORA. Of the 1944 up-regulated genes, 1887 were mapped by WebGestalt.

Category	Term ID	Biological Term	adjP (FDR)
GO: Biological Process	GO:0006955	immune response	1.29 × 10^−30^
	GO:0001775	cell activation	5.06 × 10^−28^
	GO:0007155	cell adhesion	1.26 × 10^−22^
	GO:0008283	cell population proliferation	7.80 × 10^−20^
	GO:0001816	cytokine production	1.47 × 10^−19^
	GO:0002764	immune response-regulating signaling pathway	4.94 × 10^−19^
	GO:0009607	response to biotic stimulus	1.39 × 10^−18^
	GO:0006954	inflammatory response	3.73 × 10^−18^
GO: Molecular Function	GO:0044877	protein-containing complex binding	2.18 × 10^−13^
Reactome	R-HSA-168249	innate immune system	5.02 × 10^−20^
	ETV4	ETS variant transcription factor 4	9.25 × 10^−10^
	ETS2	ETS proto-oncogene 2, transcription factor	9.25 × 10^−10^
	FOXF2	forkhead box F2	2.20 × 10^−8^
	ELF2	E74-like ETS transcription factor 2	6.25 × 10^−5^
	FOXO4	forkhead box O4	2.43 × 10^−6^
	RELA::NFKB1	RELA proto-oncogene, NF-kB subunit::nuclear factor kappa B subunit 1	1.01 × 10^−5^
	IRF1	interferon regulatory factor 1	1.03 × 10^−5^
	ZEB1	zinc finger e-box binding homeobox 1	1.42 × 10^−5^
	ETS1	ETS proto-oncogene 1, transcription factor	1.97 × 10^−5^
DisGeNET	C0017661	IgA glomerulonephritis	4.68 × 10^−8^

**Table 3:** Representative biological terms enriched in the down-regulated DEGs, as highlighted by WebGestalt ORA. Of the 2274 down-regulated genes, 2244 were mapped by WebGestalt.

Category	Term ID	Biological Term	adjP
GO: Biological Process	GO:0045333	cellular respiration	1.32 × 10^−23^
	GO:0007268	chemical synaptic transmission	4.69 × 10^−53^
	GO:0099504	synaptic vesicle cycle	8.70 × 10^−22^
	GO:0055085	transmembrane transport	8.82 × 10^−14^
GO: Cellular Component	GO:0045202	synapse	1.38 × 10^−85^
	GO:0043005	neuron projection	4.36 × 10^−54^
	GO:0036477	somatodendritic compartment	1.31 × 10^−42^
	GO:0005739	mitochondrion	1.38 × 10^−33^
	GO:0030658	transport vesicle membrane	2.45 × 10^−21^
Reactome	R-HSA-112316	neuronal system	6.89 × 10^−48^
	R-HSA-1428517	the citric acid (TCA) cycle and respiratory electron transport	1.00 × 10^−23^
Transcription Factors	REST	RE1 silencing transcription factor	1.16 × 10^−29^
	SF1	splicing factor 1	4.69 × 10^−9^
	ESRRA	estrogen-related receptor alpha	4.69 × 10^−9^
	RFX1	regulatory factor X1	2.95 × 10^−5^
	NFIL3	nuclear factor, interleukin 3 regulated	9.32 × 10^−5^

## Data Availability

All scripts necessary for conducting the analyses reported in this work are available at: https://github.com/imichalop/Meta-Analysis (accessed on 30 January 2026). Additional data concerning the results published here are available by contacting the corresponding authors.

## Supplementary Materials

The following supporting information can be downloaded at: https://www.mdpi.com/article/10.3390/biology15040345/s1, Table S1: Keywords used for search in each database; Table S2: List of 104 studies identified. A total of 91 studies were excluded from the meta-analysis and the reason for each exclusion, as well as the stage of the screening, are provided. The remaining 13 studies were included for quantitative synthesis; Table S3: Details of the results of the sample quality control (QC) per study; Table S4: Variance explained by the top three principal components of PCA analysis of the eight studies, split into 14 sub-studies, before manual sample removal, after manual sample removal, and after SVA batch correction; Figure S1: PCA plots of eight studies, split into 14 sub-studies, before manual sample removal, after manual sample removal, and after SVA batch correction; Data S1: Background gene list used for the WebGestalt over-representation analysis; Data S2: Meta-data of the included samples for meta-analysis; Data S3: The final lists of 1944 up-and 2274 down-regulated genes with adjP ≤ 0.001 in AD; Data S4: Node degree of the proteins in the STRING-based PPI network of the up- and down-regulated genes.

## Abbreviations

The following abbreviations are used in this manuscript:

AD	Alzheimer’s disease
PRISMA 2020	Perfect Reporting Items for Systemic reviews and Meta-analyses
