# Supplementary material for: Exploring Gene Expression Patterns in Alzheimer’s Disease Using a Human Microarray Data Meta-Analysis
Source: Biology (Basel). 2026 Feb 16;15(4):345. doi: 10.3390/biology15040345 (PMC12938635; doi:10.3390/biology15040345)
Supplement: Supplementary file 1 [file biology-15-00345-s001.zip › Supplementary Tables.pdf]

Table S1. Key-words used for search in each database.

Database	Search URL
GEO	https://www.ncbi.nlm.nih.gov/gds/?term=((alzheimer*[Title]+OR+alzheimer*[Description])+AND+"expression+profiling+by+array"[DataSet+Type])+AND+"Homo+sapiens"[porgn]+AND+"gse"[Filter]+AND+CEL
ArrayExpress	https://www.ebi.ac.uk/biostudies/arrayexpress/studies?facet.study_type=transcription+profiling+by+array&facet.organism=homo+sapiens&facet.file_type=cel&query="alzheimer+disease"+

Table S2. List of 104 studies identified. A total of 91 studies were excluded from the meta-analysis and the reason for each exclusion, as well as the stage of the screening, are provided. The remaining 13 studies were included for quantitative synthesis.

Database	Accession	Duplicate of	Reason for exclusion	Irrelevant metadata	Duplicate with
ArrayExpress	E-GEOD-12685	GSE12685			
ArrayExpress	E-GEOD-1297	GSE1297			
ArrayExpress	E-GEOD-16759	GSE16759			
ArrayExpress	E-GEOD-18309	GSE18309			
ArrayExpress	E-GEOD-20163	GSE20163			
ArrayExpress	E-GEOD-20164	GSE20164			
ArrayExpress	E-GEOD-21450	GSE21450			
ArrayExpress	E-GEOD-26972	GSE26972			
ArrayExpress	E-GEOD-28146	GSE28146			
ArrayExpress	E-GEOD-28379	GSE28379			
ArrayExpress	E-GEOD-29652	GSE29652			
ArrayExpress	E-GEOD-29680	GSE29680			
ArrayExpress	E-GEOD-31163	GSE31163			
ArrayExpress	E-GEOD-36980	GSE36980			
ArrayExpress	E-GEOD-37264	GSE37264			
ArrayExpress	E-GEOD-39420	GSE39420			
ArrayExpress	E-GEOD-4757	GSE4757			
ArrayExpress	E-GEOD-48350	GSE48350			
ArrayExpress	E-GEOD-5281	GSE5281			
ArrayExpress	E-GEOD-56899	GSE56899			
ArrayExpress	E-GEOD-58959	GSE58959			
ArrayExpress	E-GEOD-6276	GSE6276			
ArrayExpress	E-GEOD-6613	GSE6613			
ArrayExpress	E-GEOD-69480	GSE69480			

ArrayExpress	E-GEOD-68571	Adenocarcinoma
ArrayExpress	E-GEOD-24138	Adenovirus
GEO	GSE53890	All samples are normal
GEO	GSE131617	All samples were from AD patients, no healthy
GEO	GSE9770	All samples were from AD patients, no healthy
GEO	GSE84422	All samples were from AD patients, no healthy
ArrayExpress	E-MTAB-656	Alternative splicing, CDF not mentioned
GEO	GSE29652	Astrocyte selection
ArrayExpress	E-GEOD-5667	Atopic Dermatitis
GEO	GSE127711	Blood tissue
GEO	GSE18309	Blood tissue
GEO	GSE29680	Blood tissue
GEO	GSE56899	Blood tissue and pregnant women
GEO	GSE6613	Blood tissue, other disease (Parkinson)
GEO	GSE110226	Comparative Transcriptomics in AD with other disorders, no healthy samples
GEO	GSE222331	cultured human microglia
GEO	GSE222332	cultured human microglia
GEO	GSE79376	DU145 prostate cancer cell culture
GEO	GSE138261	Expression profiling by genome tiling array
GEO	GSE268659	Free of Dementia Histopathology
GEO	GSE6276	HEK 293 cells
GEO	GSE14429	HEK293 cells
GEO	GSE175735	human aortic endothelial cells - no AD
GEO	GSE54765	Human brain pericyte - no AD
ArrayExpress	E-GEOD-64299	Human Epithelial Cells
ArrayExpress	E-MTAB-3128	Human Keratinocytes
GEO	GSE104013	iPSC

GEO	GSE106075	iPSC
GEO	GSE117584	iPSC
GEO	GSE117585	iPSC
GEO	GSE117586	iPSC
GEO	GSE117589	iPSC
GEO	GSE143951	iPSC
GEO	GSE149599	iPSC
GEO	GSE206314	iPSC
GEO	GSE243243	iPSC
GEO	GSE43326	iPSC
GEO	GSE95810	iPSC
GEO	GSE28379	iPSC selection
GEO	GSE21779	Microcebus murinus species
ArrayExpress	E-MTAB-11983	ncRNAs (miRNAs)
GEO	GSE4757	Neuron selection
GEO	GSE66333	Neuron selection
GEO	GSE110298	Only Normal Samples
GEO	GSE30643	Other disorder (Creutzfeldt-Jakob)
GEO	GSE20163	Other disorder (Parkinson)
GEO	GSE20164	Other disorder (Parkinson)
GEO	GSE23290	Other disorder (Parkinson)
GEO	GSE34516	Other disorder (Parkinson)
GEO	GSE80244	PS1P117L cells
GEO	GSE80245	PS1P117L cells
GEO	GSE173954	Reanalysis of GSE36980
GEO	GSE69480	Selection human fibroblasts
ArrayExpress	E-MTAB-7852	Selection of human induced pluripotent stem cells (hiPS)
GEO	GSE31163	Selection of Induced pluripotent stem cells (iPSC)

ArrayExpress	E-MEXP-1913	SH-SY5Y cells		
GEO	GSE129171	SH-SY5Y cells		
GEO	GSE137202	SH-SY5Y cells		
GEO	GSE153259	SH-SY5Y cells		
GEO	GSE21450	SH-SY5Y cells		
GEO	GSE222333	Super Series containing GSE222331 and GSE222332		
GEO	GSE95843	Super Series Containing: GSE95659, GSE95810, GSE95842		
ArrayExpress	E-GEOD-64328	Super Series/ Human Epithelial Cells		
GEO	GSE58959	THP-1 cells		
GEO	GSE85238	THP-1 cells		
GEO	GSE28146		Paraffin block samples	
GEO	GSE37264			GSE37263
ArrayExpress	E-MEXP-2280			
GEO	GSE12685			
GEO	GSE1297			
GEO	GSE150696			
GEO	GSE16759			
GEO	GSE195872			
GEO	GSE26972			
GEO	GSE36980			
GEO	GSE37263			
GEO	GSE39420			
GEO	GSE48350			
GEO	GSE5281			
GEO	GSE93885			

Table S3. Details of the results of the sample quality control (QC) per study.

Studies	Initial sample number	Removed samples after QC	Sample number after QC	Removed samples after PCA	Final sample number
GSE48350	253	43 (GSM1176213, GSM1176216, GSM1176218, GSM1176219, GSM1176223, GSM1176224, GSM1176228, GSM1176230, GSM1176235, GSM1176244, GSM1176260, GSM1176261, GSM1176268, GSM1176272, GSM300168, GSM300169, GSM300170, GSM300171, GSM300172, GSM300185, GSM300186, GSM300189, GSM300190, GSM300191, GSM300202, GSM300212, GSM300213, GSM300227, GSM300228, GSM300243, GSM300250, GSM300251, GSM300255, GSM300256, GSM300260, GSM300276, GSM300278, GSM300282, GSM300326 and removed duplicates GSM1176197, GSM1176215, GSM1176233, GSM1176257)	210	36 from Super Frontal Gyrus (GSM1176271, GSM1176273, GSM300176, GSM300207, GSM300211, GSM300221, GSM300225, GSM300229, GSM300233, GSM300241, GSM300245, GSM300247, GSM300254, GSM300264, GSM300266, GSM300270, GSM300274, GSM300292, GSM300296, GSM300299, GSM300303, GSM300307, GSM300315, GSM300319, GSM300323, GSM300331, GSM300341, GSM1176255, GSM1176256, GSM1176258, GSM1176262, GSM1176263, GSM1176265, GSM1176267, GSM1176269, GSM1176270)<br>37 from Post Central Gyrus (GSM1176254, GSM300166, GSM300175, GSM300183, GSM300200, GSM300210, GSM300216, GSM300246, GSM300249, GSM300253, GSM300259, GSM300263, GSM300265, GSM300269, GSM300273, GSM300281, GSM300283, GSM300295, GSM300302, GSM300310, GSM300314, GSM300322, GSM300340, GSM1176231, GSM1176237, GSM1176238, GSM1176239, GSM1176240, GSM1176242, GSM1176243, GSM1176245, GSM1176246, GSM1176247, GSM1176249, GSM1176250, GSM1176252, GSM1176253)<br>14 from Hippocampus (GSM300325, GSM300333, GSM1176222, GSM1176225, GSM1176227, GSM1176229, GSM300187,	109

				GSM300193, GSM300197, GSM300219, GSM300272, GSM300290, GSM300301, GSM300305)<br>14 from Entorhinal Cortex (GSM300332, GSM1176196, GSM1176205, GSM1176209, GSM300196, GSM300208, GSM300214, GSM300234, GSM300248, GSM300252, GSM300261, GSM300267, GSM300300, GSM300328)	
GSE39420	21	6 (GSM967918, GSM967920, GSM967924, GSM967926, GSM967928, GSM967935)	15	1 (GSM967927)	14
GSE36980	80	10 (GSM907793, GSM907795, GSM907797, GSM907798, GSM907815, GSM907838, GSM907857, GSM907860, GSM907866, GSM4764672) and 17 were additionally removed due to not being purely AD or no dementia samples (GSM907792, GSM907803, GSM907809, GSM907811, GSM907812, GSM907818, GSM907822, GSM907824, GSM907825, GSM907830, GSM907836, GSM907842, GSM907845, GSM907856, GSM907861, GSM907863, GSM907868)	53	7 from Temporal Cortex (GSM907826, GSM907828, GSM907832, GSM907833, GSM907841, GSM907844, GSM907846)<br>1 from Hippocampus (GSM907869)<br>7 from Frontal Cortex (GSM907801, GSM907802, GSM907817, GSM907819, GSM907820, GSM907821, GSM907823)	38
E-MEXP-2280	12	3 (050705MJA_U133_2.0_IB09, 050705MJA_U133_2.0_IB59, 050705MJA_U133_2.0_IB10)	9	0	9
GSE16759	8	1 (GSM420149)	7	0	7
GSE1297	31	4 (GSM21205, GSM21207, GSM21213, GSM21218)	27	17 (GSM21230, GSM21231, GSM21232, GSM21233, GSM21203, GSM21204, GSM21206, GSM21208, GSM21217, GSM21219, GSM21221, GSM21222, GSM21224, GSM21225, GSM21227, GSM21228, GSM21229)	10
GSE5281	161	121 (GSM119615 up to GSM119623, GSM119626 up to GSM119632,	40	1 from Primary Visual Cortex (GSM119681)	39

		GSM119634 up to GSM119652, GSM119655, GSM119656, GSM119659 up to GSM119663, GSM119665 up to GSM119669, GSM119671 up to GSM119676, GSM119679, GSM119682 up to GSM119686, GSM119688, GSM238763, GSM238790 up to SM238799, GSM238801 up to GSM238803, GSM238805 up to GSM238807, GSM238809 up to GSM238813, GSM238815, GSM238816, GSM238819 up to GSM238825, GSM238827, GSM238834, GSM238837 up to GSM238840, GSM238845, GSM238847, GSM238848, GSM238855, GSM238858, GSM238863, GSM238865, GSM238867, GSM238870, GSM238875, GSM238877, GSM238941 up to GSM238944, GSM238946 up to GSM238955, GSM238963)			
GSE12685	14	2 (GSM318212, GSM318321)	12	1 (GSM318211)	11
GSE195872*	13	All samples (GSM5855442, GSM5855443, GSM5855446, GSM5855449, GSM5855450, GSM5855453, GSM5855457, GSM5855458, GSM5855461, GSM5855465, GSM5855466, GSM5855469, GSM5855473)	0		0
GSE150696*	42	All samples (GSM4556850, GSM4556851, GSM4556852, GSM4556853, GSM4556854, GSM4556855, GSM4556856, GSM4556857, GSM4556858, GSM4556859, GSM4556860, GSM4556861,	0		0

		GSM4556862, GSM4556863, GSM4556864, GSM4556865, GSM4556866, GSM4556867, GSM4556868, GSM4556869, GSM4556870, GSM4556872, GSM4556875, GSM4556876, GSM4556877, GSM4556878, GSM4556879, GSM4556880, GSM4556881, GSM4556882, GSM4556883, GSM4556884, GSM4556885, GSM4556886, GSM4556887, GSM4556888, GSM4556889, GSM4556890, GSM4556891, GSM4556892, GSM4556893, GSM4556894)		
GSE93885*	18	11 (GSM2464460, GSM2464461, GSM2464462, GSM2464463, GSM2464466, GSM2464467, GSM2464468, GSM2464469, GSM2464471, GSM2464476, GSM2464477)	7	7

*Ultimately not included in subsequent analyses, as all control or all samples were discarded or <3 control samples remained for analysis

Table S4. Variance explained by the top three principal components of PCA analysis of the eight studies, split into 14 sub-studies, before manual sample removal, after manual sample removal, and after SVA batch-correction.

Study/SubStudy	PCA before manual sample removal				PCA after manual sample removal				PCA after SVA			
	PCA1	PCA2	PCA3	Top 3 PCA variance	PCA1	PCA2	PCA3	Top 3 PCA variance	PCA1	PCA2	PCA3	Top 3 PCA variance
E-MEXP-2280	0.34	0.27	0.11	0.72					0.32	0.23	0.17	0.72
GSE1297	0.28	0.16	0.07	0.51	0.5	0.11	0.1	0.71	0.44	0.18	0.14	0.76
GSE5281_PCC	0.28	0.21	0.13	0.62					0.65	0.19	0.16	1
GSE5281_PVC	0.35	0.14	0.11	0.6	0.35	0.16	0.13	0.64	0.5	0.26	0.24	1
GSE12685	0.3	0.29	0.07	0.66	0.41	0.1	0.1	0.61	0.63	0.22	0.15	1
GSE16759	0.39	0.18	0.15	0.72								
GSE36980_FrontCort	0.18	0.11	0.09	0.38	0.21	0.15	0.1	0.46	0.36	0.13	0.12	0.61
GSE36980_Hip	0.3	0.16	0.12	0.58	0.34	0.17	0.12	0.63	0.31	0.18	0.13	0.62
GSE36980_TempCort	0.15	0.12	0.08	0.35	0.21	0.14	0.11	0.46	0.24	0.12	0.09	0.45
GSE39420	0.56	0.1	0.07	0.73	0.53	0.11	0.08	0.72	0.5	0.1	0.08	0.68
GSE48350_EnthCortx	0.44	0.07	0.05	0.56	0.45	0.07	0.06	0.58	0.44	0.08	0.06	0.58
GSE48350_Hip	0.28	0.12	0.08	0.48	0.35	0.1	0.08	0.53				
GSE48350_PostCentGyrus	0.34	0.09	0.06	0.49	0.39	0.09	0.08	0.56	0.32	0.05	0.04	0.41
GSE48350_SuperFroGyrus	0.33	0.12	0.06	0.51	0.39	0.13	0.07	0.59	0.4	0.08	0.06	0.54
