# Supplementary figures and images for: Exploring Gene Expression Patterns in Alzheimer’s Disease Using a Human Microarray Data Meta-Analysis
Source: Biology (Basel). 2026 Feb 16;15(4):345. doi: 10.3390/biology15040345 (PMC12938635; doi:10.3390/biology15040345)

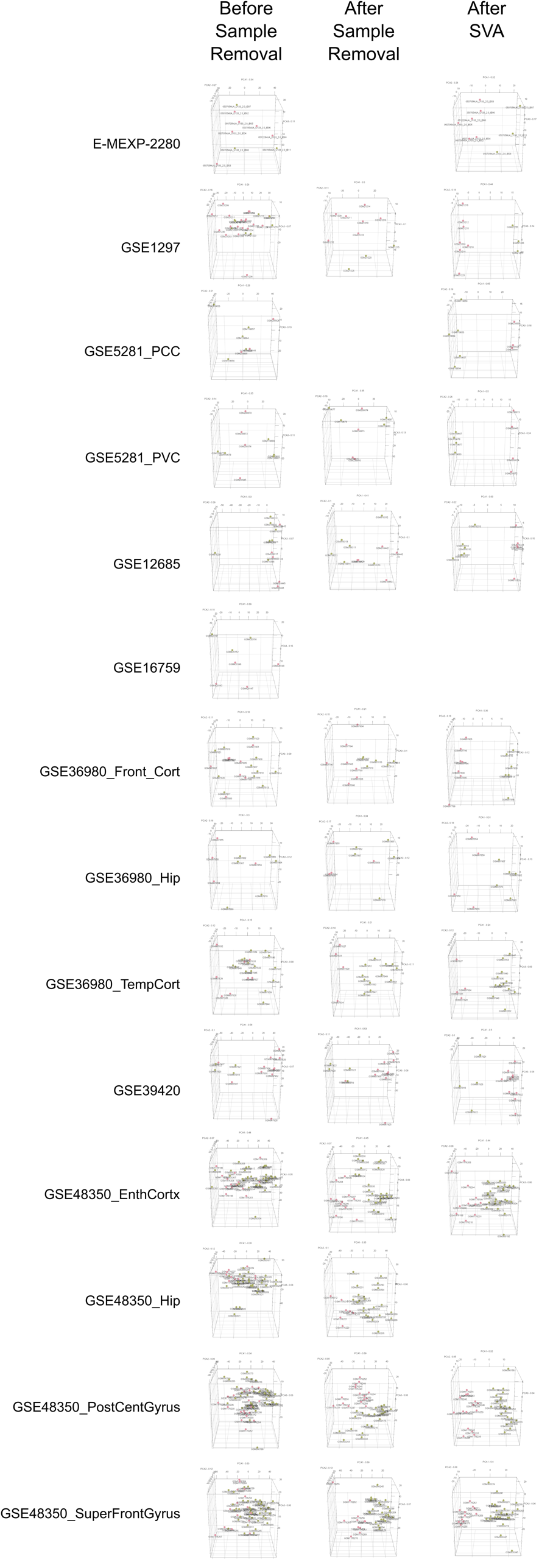

Supplement: Supplementary file 1 [file biology-15-00345-s001.zip › FigureS1.jpg]
